# Different functional lung-sparing strategies and radiotherapy techniques for patients with esophageal cancer

**DOI:** 10.3389/fonc.2022.898141

**Published:** 2022-08-26

**Authors:** Pi-Xiao Zhou, Rui-Hao Wang, Hui Yu, Ying Zhang, Guo-Qian Zhang, Shu-Xu Zhang

**Affiliations:** Radiotherapy Center, Affiliated Cancer Hospital and Institute of Guangzhou Medical University, Guangzhou, China

**Keywords:** esophageal cancer, four-dimensional CT (4D-CT), functional lung, intensity-modulated radiotherapy (IMRT), volumetric-modulated arc therapy (VMAT)

## Abstract

**Background:**

Integration of 4D-CT ventilation function images into esophageal cancer radiation treatment planning aimed to assess dosimetric differences between different functional lung (FL) protection strategies and radiotherapy techniques.

**Methods:**

A total of 15 patients with esophageal cancer who had 4D-CT scans were included. Lung ventilation function images based on Jacobian values were obtained by deformation image registration and ventilation imaging algorithm. Several different plans were designed for each patient: clinical treatment planning (non-sparing planning), the same beam distribution to FL-sparing planning, three fixed-beams FL-sparing intensity-modulated radiation therapy (IMRT) planning (5F-IMRT, 7F-IMRT, 9F-IMRT), and two FL-sparing volumetric modulated arc therapy (VMAT) planning [1F-VMAT (1-Arc), 2F-VMAT (2-Arc)]. The dosimetric parameters of the planning target volume (PTV) and organs at risk (OARs) were compared and focused on dosimetric differences in FL.

**Results:**

The FL-sparing planning compared with the non-sparing planning significantly decreased the FL-D_mean_, V_5-30_ and Lungs-D_mean_, V_10-30_ (V_x_: volume of receiving ≥X Gy), although it slightly compromised PTV conformability and increased Heart-V_40_ (P< 0.05). The 5F-IMRT had the lowest PTV-conformability index (CI) but had a lower Lungs and Heart irradiation dose compared with those of the 7F-IMRT and 9F-IMRT (P< 0.05). The 2F-VMAT had higher PTV-homogeneity index (HI) and reduced irradiation dose to FL, Lungs, and Heart compared to those of the 1F-VMAT planning (P< 0.05). The 2F-VMAT had higher PTV conformability and homogeneity and decreased FL-D_mean_, V_5_-_20_ and Lungs-D_mean_, V_5_-_10_ but correspondingly increased spinal cord-D_mean_ compared with those of the 5F-IMRT planning (P< 0.05).

**Conclusion:**

In this study, 4D-CT ventilation function image-based FL-sparing planning for esophageal cancer can effectively reduce the dose of the FL. The 2F-VMAT planning is better than the 5F-IMRT planning in reducing the dose of FL.

## Introduction

Esophageal cancer (EC) is a malignant tumor originating from the mucosal epithelium of the esophagus, which is one of the common gastrointestinal malignancies and the sixth most common cancer-related cause of death globally (1). In China, the incidence of EC is relatively high, and the number of new cases and deaths each year accounts for 53.7% and 55.7% of the global total, respectively (2). Radiotherapy is one of the effective treatment options for patients with EC (1). However, radiation pneumonitis (RP) is a common and potentially fatal toxicity reaction to radiation therapy for thoracic tumors such as EC, with a G2+ RP incidence of 6%–25% (3). It may lead to pulmonary fibrosis and lung function compromise and, in severe cases, may cause death due to respiratory distress (4). It can also limit the improvement of the clinical prescription dose, which may affect the efficacy and prognosis. Although with the advancement of radiotherapy technology, intensity-modulated radiation therapy (IMRT) compared with three-dimensional conformal radiation therapy (3D-CRT) improved the target conformability and decreased the organ at risk (OAR) radiation dose. However, according to a meta-analysis, IMRT did not significantly reduce the incidence of RP in EC compared with 3D-CRT (5). Furthermore, volumetric modulated arc therapy (VMAT) is a more advanced radiotherapy technique than IMRT that can further reduce the dose of OARs (6).

Previous studies have shown that the occurrence of RP was related to the dose and volume of lung irradiation and that there was heterogeneity in the response of lung tissue to radiation in different functional states (7, 8). In addition, the functional subunits are not uniformly distributed owing to organ structure or disease (e.g., lung and liver) (9). However, conventional anatomical CT planning does not take the heterogeneity of lung function distribution into consideration, but the 3D map of functional lung (FL; high functional state) distribution identified by FL imaging can be integrated into radiotherapy planning (10). Faught et al. (11) used the normal tissue concurrent probability (NTCP) model to predict the incidence of RP in the FL-sparing planning group. The results showed that the FL-sparing planning decreased the incidence of grade 2+ and 3+ RP in lung cancer patients by 7.1% and 4.7% compared with the conventional anatomical CT planning, respectively. Moreover, the FL dose-volume parameters (e.g., functional lung mean dose (f-MLD), volume of functional lung receiving ≥ 20Gy (fV_20_)) can more accurately predict the incidence of RP than anatomical lung parameters (MLD, V_20_) (12). Currently, several ongoing clinical trials are investigating the clinical value of using FL-sparing planning-guided radiotherapy to reduce RP (e.g., NCT02308709, NCT02843568, NCT04676828) (13, 14).

Previous functional imaging was commonly used to assess tumor heterogeneity, evaluate efficacy, and predict prognosis, and fewer studies have extended to evaluate heterogeneity of lung function distribution (15). At present, FL imaging modalities include four-dimensional computed tomography (4D-CT), dual-energy CT, magnetic resonance image (MRI), single-photon emission computed tomography (SPECT), and positron emission tomography (PET) (16). 4D-CT imaging has been routinely used in lung cancer radiotherapy workflow for respiratory motion management and individual target area delineation (ITV). It also has the advantages of high-speed scanning, higher resolution, lower cost, and the ability to acquire 3D distribution images of lung ventilation function without relying on additional functional imaging equipment and methods (17, 18). Studies have validated the accuracy of 4D-CT lung ventilation function imaging by correlating it with clinical pulmonary function test (PFT) and nuclear medicine (SPECT/CT, PET/CT) ventilation function imaging, and both results demonstrated a good correlation (18, 19). Pinder-Arabpour et al. (20) demonstrated the significant heterogeneity in the distribution of lung ventilation function in EC patients for the first time in 2019. Currently, FL imaging studies have not been applied to radiotherapy for EC separately. Therefore, this study will investigate the dosimetric value of different protection strategies and radiotherapy techniques for protecting FL based on the 4D-CT lung ventilation function image in EC patients.

## Materials and methods

### Patient population

Patients with EC scanned with 4D-CT and treated with radiotherapy in our hospital from 1 October 2021 to 20 February 2022 were selected for this study. Inclusion criteria were as follows: 1) The planning target volume (PTV) was located in the thoracic esophagus (including upper thoracic, middle thoracic, and lower thoracic); 2) Patients had not received previous radiotherapy to the thoracic; 3) There was no restriction on the type of radiotherapy that the patient received (neoadjuvant, adjuvant, or definitive radiotherapy). 4) 4D-CT scanning data were available.

### Contrast-enhanced CT and 4D-CT scanning

All patients were immobilized in the supine position using a thermoplastic mold, and enhanced CT was performed by Brilliance Big Bore scanner (Phillips Healthcare, USA). The scanning range was from the upper edge of the first cervical vertebra (C1) or the lower edge of the seventh cervical vertebra (C7) to the upper abdomen, with the following parameters: voltage 120 kVp, current 300 mA, and slice thickness/spacing of 3–5 mm. The 4D-CT scans were performed after the enhanced CT was completed, and a marker module had been placed on the abdomen where the respiratory magnitude was most apparent (no marker was implanted). Using Varian’s Real-Time Position Management (RPM) System to monitor the patient’s respiratory waveform, 4D-CT scanning was performed under free breathing without any breathing control. The scanning parameters were the same as above, and the CT data were reconstructed into 10 respiratory phases using the respiratory curve after completion. The enhanced CT and 4D-CT data were then transmitted to the Pinnacle^3^ (Version: 9.10, Philips Healthcare, USA) and Eclipse (Version 15.1, Varian Medical Systems, USA) treatment planning systems (TPSs), respectively.

### 4D-CT ventilation function imaging

**Figure 1:**
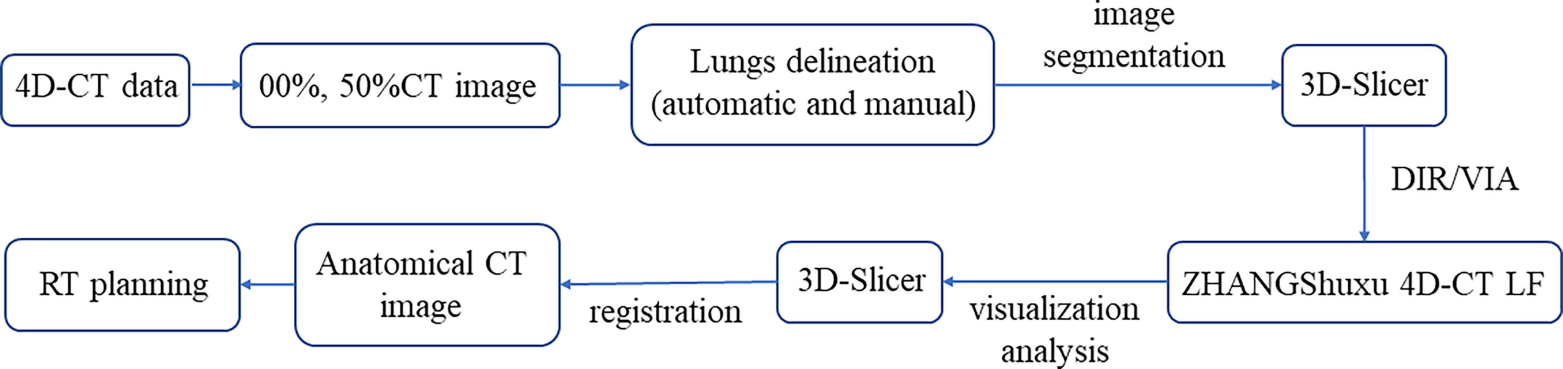
Flowchart of the functional lung obtained from 4D-CT. DIR, deformation image registration; VIA, ventilation imaging algorithm.

Lung ventilation function images are primarily obtained through two steps. The first is deformation image registration (DIR) and the second is the ventilation imaging algorithm (VIA) (21). In this study, the end-inspiratory CT image (00%) was used as the reference image, and the end-expiratory CT image (50%) was used as a variable image for the registration and calculation. The combination of automatic and manual (removing redundant main bronchi, correcting incorrectly delineated areas, and completing lung tissue) delineation was used to generate the whole lung (Lungs) area on 00%, 50%, and average intensity projection (AIP) CT images in Eclipse. Export to 3D-Slicer software (Version 4.11.20200930, http://www.slicer.org), performing image segmentation to form the corresponding VTK files (00%.VTK, 50%.VTK). Then, the VTK files were imported into our self-developed ventilation imaging software (ZHANGShuxu 4D-CT LF, V1.0) for DIR and quantitative calculations (22, 23). Jacobian determinant of deformation was utilized to measure the corresponding lung volume changes with the two CT images (23, 24). Finally, the Jacobian data and AIP images files were imported into 3D-Slicer for visualization and quantitative analysis of lung ventilation function (**Figure 1**). When Jacobian = 1, it indicates no volume change in the corresponding area of two images. When Jacobian<1, the related volume shrinks compared to the reference image (24).

### Target and organ at risk delineation

**Figure 2:**
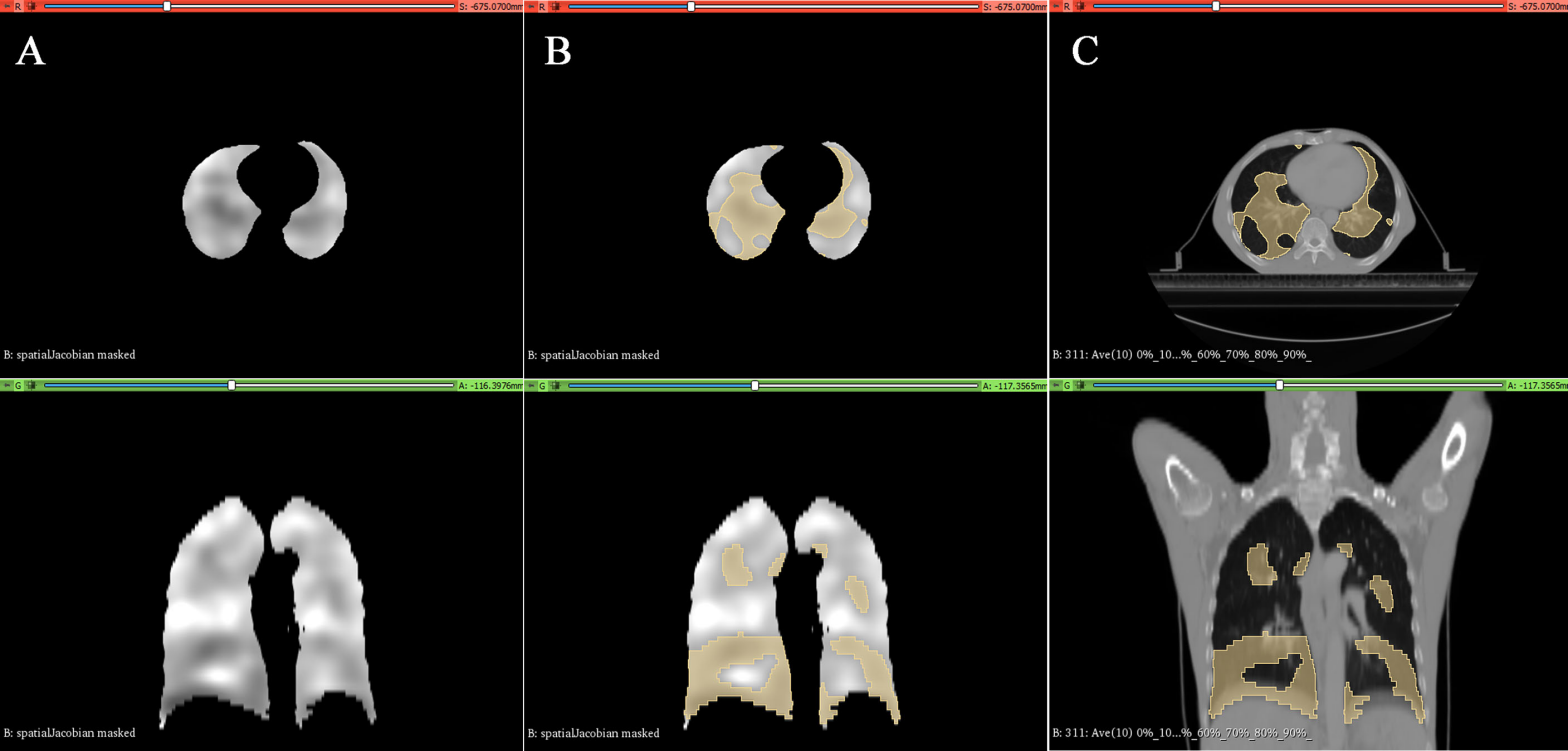
Typical lung ventilation function images generated in 3D-Slicer software. **(A)** Grayscale image containing the Jacobian value. **(B)** Defined regions of Jacobian value ≤0.8 (e.g., functional lung). **(C)** Distribution of functional lungs in the esophageal cancer patient’s anatomical CT.

The tumor target area and OARs were delineated on Pinnacle^3^ by an experienced radiation oncologist of our hospital according to the Chinese EC radiotherapy guidelines and the International Commission on Radiation Units and Measurements (ICRU) Report 62 (4, 25) and then reviewed by a senior radiation oncologist. Gross tumor target volume (GTV) was defined as the primary tumor/visible esophageal lesion (GTVp) and metastatic lymph nodes (GTVn). The clinical target volume (CTV) was defined as an 8-mm expansion of the GTV in the anterior–posterior, left–right, and superior–inferior directions. PTV is defined as CTV with 5-mm expansion in all directions. Because the esophagus is close to the Spinal cord, Heart, and surrounded by Lungs, these organs are the significant OARs. In this study, the FL is another essential OAR. Based on our prior research results, regions with a Jacobian value ≤0.8 were defined as FL (26). The 3D distribution map of the FL was exemplified in **Figure 2**.

### Radiotherapy planning

Radiotherapy planning was designed for each patient on the Pinnacle^3^ 9.10, including a conventional anatomical CT treatment planning (without consideration of FL, non-sparing planning), as well as the same beam distribution FL-sparing planning, three fixed-beams FL-sparing IMRT planning [5F-IMRT (0°, 72°, 144°, 216°, 288°), 7F-IMRT (0°, 50°, 100°, 150°, 210°, 260°, 310°), 9F-IMRT (0°, 40°, 80°, 120°, 160°, 200°, 240°, 280°, 320°)], and two FL-sparing VMAT planning [1F-VMAT (1-Arc), 2F-VMAT (2-Arc)]. The non-sparing planning was accomplished through the same group of experienced physicists and radiation oncologists in consultation. The linear accelerator energy was 6 MV, and the radiation dose was 1.8–2.2 Gy/20–30 (fractions), five times a week. Prescription dose lines contain at least 95% of the PTV, and the hot spot (≤110% prescription dose) could not fall on the OARs. The FL-sparing planning for EC was consistent with the clinical treatment planning regarding prescription dose, target area dose requirements, OAR dose limitations, and weights while only requiring additional dose limitations for the FL. The PTV gave the highest priority (100%), and the FL was as low as possible under the condition that the doses of the PTV and OARs meet clinical request. The OAR dose-limitation schemes are shown in **Table 1**.

**Table 1:** The dose-volume restrictions of organs at risk (OARs).

OARs	Restrictions
Lungs	V_5_<65%, V_20_<30%, V_30_<20%
Heart	V_40_<40%
Spinal cord	D_max_<45Gy
Functional lung	V_10_<20%, V_20_<10%, V_30_<5%

### Planning evaluation

Dose-volume histograms (DVHs) were analyzed for the PTV and OARs. PTV evaluated its conformability index (CI) and homogeneity index (HI). CI is defined to assess the conformity of the prescribed dose distribution (27).


$$CI=\frac{V_{P,ref}}{V_{P}}\times \frac{V_{P,ref}}{V_{ref}}$$

V_P, ref_, V_P_, V_ref_ represented the volume of PTV surrounded by the prescription dose line, the volume of PTV, and the volume surrounded by the prescription dose line, respectively. The CI ranges from 0 to 1, and closer to 1 means better conformability of the PTV. HI was used to evaluate the uniformity of prescription dose distribution in PTV and was calculated by the following equation:


$$HI=\frac{D_{5\%}}{D_{95\%}}$$

D_5%_, D_95%_ represented the dose received 5%, 95% volume of the PTV, respectively. The closer the HI to 1, the better homogeneity of the PTV. MLD (lung mean dose), V_5_ (V_x_, volume of receiving dose ≥ × Gy), V_10_, V_20_, and V_30_ were evaluated for the whole lung, FL, and high FL, MHD (heart mean dose), V_5_, V_10_, V_20_, V_30_, and V_40_ for the heart, and D_max_ (maximum dose) and D_mean_ for the spinal cord.

### Statistical methods

The measurements were described by mean ± standard deviation (SD); paired t-test was conducted to compare the dose-volume parameters of PTV and OAR difference between different groups. Statistical analysis was performed with SPSS 25.0 (IBM SPSS Statistics, USA), and P< 0.05 was considered statistically significant.

## Results

A total of 15 patients were included, 14 men and 1 woman, with a mean age of 57.2 years (48–68 years). The mean volume of CTV was 319.7 ± 127.4 cm^3^. More than half of the patients had PTV in the upper and middle thoracic esophagus. Detailed clinical information of the patients is shown in **Table 2**.

**Table 2:** Detailed clinical information of the patients included in the study.

No. of patients	15
**Mean age (range)**	57.2 (48–68) years
**Gender, n (%)**	
Men	14 (93.3%)
Women	1 (6.7%)
**Histology, n (%)**	
Squamous cell carcinoma	13 (86.7%)
Small-cell carcinoma	2 (13.3%)
**Target location (PTV), n (%)**	
U+M	7 (46.7%)
M	3 (20%)
M+L	3 (20%)
L	1 (6.7%)
U+M+L	1 (6.7%)
**Mean CTV (range, cm^3^)**	319.7 (161.7–558.3)
**Mean prescription dose (PTV, range)**	48.5 (36–60.2) Gy
**Clinical treatment planning, n (%)**	
IMRT	12 (80%)
VMAT	3 (20%)

SD, standard deviation; PTV, planning target volume; CTV, clinical target volume; U, upper thoracic; M, middle thoracic; L, lower thoracic.

### Comparison of non-sparing and functional lung-sparing planning

The PTV and OAR dosimetric differences of the non-sparing planning and FL-sparing planning with consistent beam arrangement are listed in **Table 3**. Compared with the non-sparing planning, the FL-sparing planning has a slighter lower CI (0.662 ± 0.098 vs. 0.692 ± 0.083, P = 0.024) and a similar HI (1.144 ± 0.064 vs. 1.142 ± 0.078, P > 0.05), indicating a slightly lower conformity dose distribution to the PTV. In general, both plans maintained a good coverage of the PTV.

**Table 3:** Dosimetric parameter comparison for PTV and OARs in different FL-sparing IMRT and VMAT planning.

OARs	Non-sparing planning	FL-sparing planning	P value
PTV			
D_max_ (Gy)	56.98 ± 8.46	58.26 ± 9.01	0.005
D_mean_ (Gy)	52.42 ± 7.98	52.60 ± 8.01	0.178
CI	0.692 ± 0.083	0.662 ± 0.098	0.024
HI	1.142 ± 0.078	1.144 ± 0.064	0.806
FL			
D_mean_ (Gy)	7.38 ± 2.95	5.94 ± 2.26	<0.001
V_5_ (%)	39.68 ± 16.32	37.71 ± 14.82	0.041
V_10_ (%)	25.03 ± 10.24	15.79 ± 7.79	<0.001
V_20_ (%)	11.00 ± 6.87	6.19 ± 3.72	<0.001
V_30_ (%)	4.70 ± 4.18	3.42 ± 2.49	0.033
Lungs			
D_mean_ (Gy)	9.91 ± 2.58	9.14 ± 2.56	<0.001
V_5_ (%)	50.84 ± 11.72	49.58 ± 11.82	0.117
V_10_ (%)	35.09 ± 8.11	29.70 ± 7.52	<0.001
V_20_ (%)	16.40 ± 5.86	13.82 ± 5.52	0.009
V_30_ (%)	7.45 ± 4.37	6.85 ± 3.72	0.039
Heart			
D_mean_ (Gy)	17.12 ± 10.53	17.55 ± 10.68	0.298
V_5_ (%)	61.07 ± 35.27	60.96 ± 35.38	0.771
V_10_ (%)	53.46 ± 34.24	53.14 ± 34.46	0.766
V_20_ (%)	40.92 ± 29.67	40.00 ± 27.45	0.562
V_30_ (%)	22.47 ± 16.02	24.46 ± 17.11	0.078
V_40_ (%)	11.67 ± 9.91	13.57 ± 11.57	0.035
Spinal cord			
D_max_ (Gy)	38.82 ± 4.67	37.69 ± 5.87	0.600
D_mean_ (Gy)	11.99 ± 9.05	12.47 ± 9.53	0.142

Mean ± SD; P value was calculated by paired t-test. PTV, planning target volume; OARs, organs at risk; FL, functional lung; Dmax, maximum dose; Dmean, mean dose; Vx, volume of receiving = X Gy.

**Figure 3:**
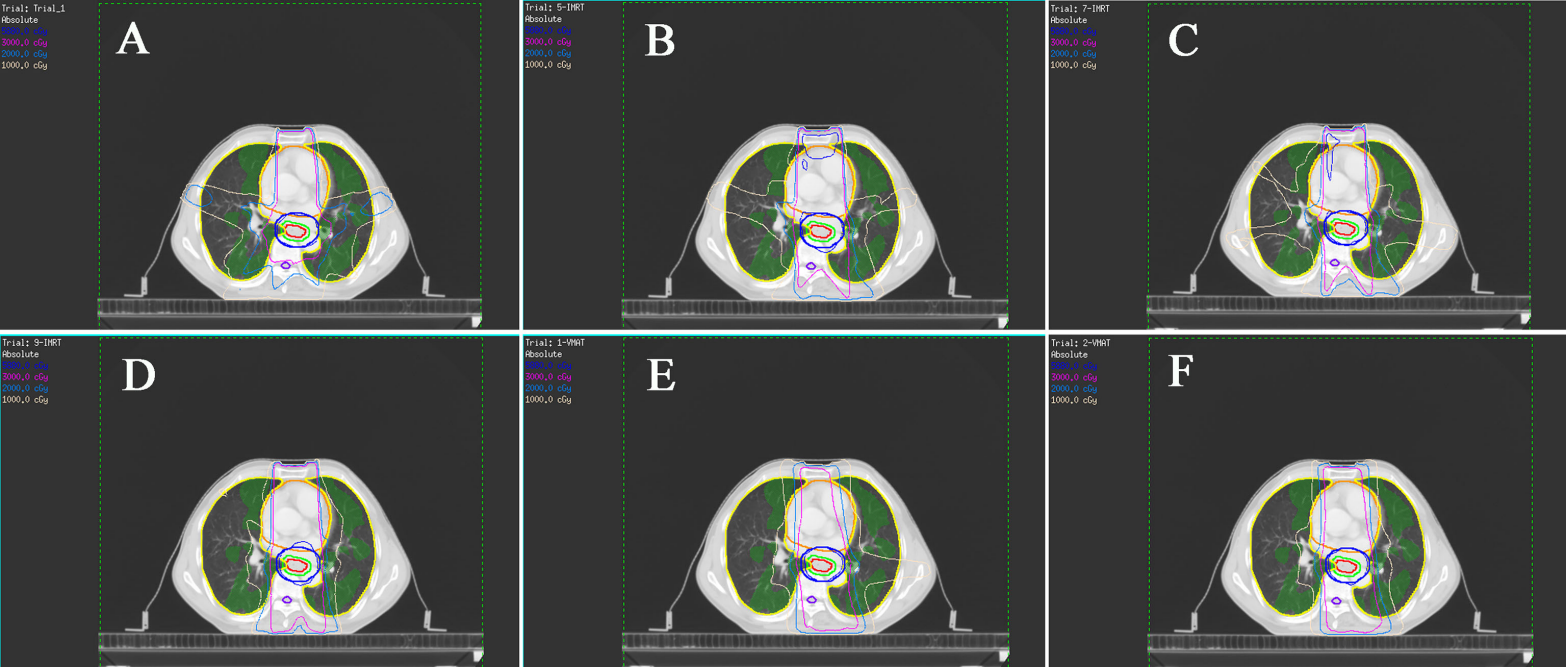
Typical isodose dose distribution map of the radiotherapy planning for esophageal cancer patients. The functional lung (FL) are green areas. **(A)** Non-sparing planning (five-field fixed-beam IMRT); **(B)** five-field fixed-beam functional lung-sparing IMRT planning (5F-IMRT); **(C)** seven-field fixed-beam FL-sparing IMRT planning (7F-IMRT); **(D)** nine-field fixed-beam FL-sparing IMRT planning (9F-IMRT); **(E)** one-arc FL-sparing VMAT planning (1F-VMAT). **(F)** two-arc FL-sparing VMAT planning (2F-VMAT).

The dosimetric parameters of FL are also listed in **Table 3**, and the typical planning and dose-volume histogram for FL are shown in **Figure 3**. Compared with those in the non-sparing planning group, the FL-V_5_, V_10_, V_20_, V_30_, and D_mean_ were significantly reduced in the FL-sparing planning group (P< 0.05). The dosimetric parameters of reduction are presented as follows: 1.97% for FL-V_5_ (non-sparing vs. FL-sparing: 39.68% ± 16.32% vs. 37.71% ± 14.82%, P = 0.041), 9.24% for FL-V_10_ (25.03% ± 10.24% vs. 15.79% ± 7.79%, P< 0.001), 4.81% for FL-V_20_ (11.00% ± 6.87% vs. 6.19% ± 3.72, P< 0.001), 1.28% for FL-V_30_ (4.70% ± 4.18% vs. 3.42% ± 2.49%, P = 0.033), and 1.44 Gy for FL-D_mean_ (7.38 ± 2.95 Gy vs. 5.94 ± 2.26 Gy, P< 0.001).

The dosimetric parameters for the other OARs are also presented in **Table 3**. According to the results, the dose limitation for one of the OARs will inevitably increase the dose to another. The D_mean_, V_10_, V_20_, and V_30_ of the Lungs are significantly decreased in the FL-sparing planning, which may be caused by the dose restriction of the FL. The FL-sparing planning had a statistically significant increase in Heart-V_40_ compared to that of the non-sparing planning. However, the D_max_ and D_mean_ of the spinal cord showed no significant difference in these two plans.

### Comparison of 5F-IMRT, 7F-IMRT, and 9F-IMRT planning

Different FL-sparing IMRT plannings were then investigated to evaluate their value in reducing the dose of the FL (**Supplementary Table S1**). Regarding the conformal and uniform dose distribution of the PTV, it was observed that 5F-IMRT had the lowest CI 5F-IMRT (five-field fixed-beam functional lung-sparing IMRT planning) vs. 7F-IMRT (seven-field fixed-beam functional lung-sparing IMRT planning)/9F-IMRT (nine-field fixed-beam functional lung-sparing IMRT planning): 0.647 vs. 0.670/0.681, P< 0.05; 7F-IMRT vs. 9F-VMAT: P > 0.05), while D_max_, D_mean_, and HI were not found to be statistically different between the three plans. The 5F-IMRT compared with the 7F-IMRT had lower Lungs-D_mean_, Heart-V_5_, and V_10_ (P< 0.05). The 5F-IMRT compared with the 9F-IMRT had higher FL-V_30_ but lower Heart-V_5_ (P< 0.05). The 7F-IMRT compared with the 9F-IMRT had higher Lungs-D_mean_ and V_10_ (P< 0.05). There was no significant difference in spinal cord-D_mean_ and D_max_ between the three plans (P > 0.05).

### Comparison of 1F-VMAT and 2F-VMAT planning

The differences in dose reduction for FL between the different FL-sparing VMAT planning were also explored (**Supplementary Table S2**). The 2F-VMAT significantly decreased the PTV-HI compared with the 1F-VMAT planning, indicating that the 2F-VMAT had higher PTV homogeneity. At the same time, D_max_, D_mean_, and CI of the PTV were not statistically different between the two plans (P > 0.05). The 2F-VMAT reduced FL-D_mean_, V_10_, Lungs-V_10_, and Heart-V_20_ compared to the 1F-VMAT planning (P< 0.05).

### Comparison of functional lung-sparing IMRT and VMAT planning

Subsequently, the dosimetric differences between different FL-sparing IMRT and VMAT planning were further analyzed (**Table 4**). The dosimetric differences in PTV and OARs between the 5F-IMRT and 2F-VMAT planning were selected for comparison on the premise that the PTV dose meets the clinical requirements, with a preference for low Lungs, Heart, and spinal cord irradiated doses and followed by low FL irradiated doses. The 2F-VMAT had higher target area conformability and homogeneity compared to the 5F-IMRT planning. The 2F-VMAT decreased FL-D_mean_, V_5_, V_10_, V_20_, and Lungs-D_mean_, V_5_, V_10_ but correspondingly increased spinal cord-D_mean_ (P< 0.05) compared with the 5F-IMRT planning. The irradiated dose of the Heart was not statistically different between the two plans (P > 0.05).

**Table 4:** Dosimetric comparison in PTV and OARs between non-sparing (clinical treatment) planning and consistent beam directions of FL-sparing planning.

OARs	5F-IMRT	2F-VMAT	P value
PTV			
D_max_ (Gy)	58.19 ± 8.83	56.77 ± 8.75	<0.001
D_mean_ (Gy)	52.54 ± 7.99	52.43 ± 7.96	0.225
CI	0.647 ± 0.106	0.711 ± 0.113	0.002
HI	1.152 ± 0.076	1.131 ± 0.071	0.019
FL			
D_mean_ (Gy)	6.04 ± 2.32	5.64 ± 2.01	0.004
V_5_ (%)	37.61 ± 14.95	34.78 ± 13.32	0.027
V_10_ (%)	16.55 ± 7.65	13.27 ± 5.99	0.002
V_20_ (%)	6.52 ± 3.76	5.99 ± 3.57	0.037
V_30_ (%)	3.61 ± 2.53	3.22 ± 2.29	0.097
Lungs			
D_mean_ (Gy)	9.18 ± 2.63	8.69 ± 2.43	0.004
V_5_ (%)	50.44 ± 13.05	46.03 ± 9.80	0.002
V_10_ (%)	30.51 ± 8.82	27.07 ± 7.52	0.001
V_20_ (%)	13.71 ± 5.43	13.20 ± 5.62	0.335
V_30_ (%)	6.86 ± 3.56	6.46 ± 3.27	0.104
Heart			
D_mean_ (Gy)	17.38 ± 10.30	17.15 ± 10.34	0.302
V_5_ (%)	60.88 ± 35.20	61.51 ± 35.53	0.207
V_10_ (%)	53.10 ± 34.06	53.89 ± 33.48	0.518
V_20_ (%)	38.34 ± 25.07	39.56 ± 26.96	0.277
V_30_ (%)	23.80 ± 16.08	22.59 ± 15.86	0.050
V_40_ (%)	13.61 ± 11.30	12.17 ± 10.41	0.160
Spinal cord			
D_max_ (Gy)	40.01 ± 4.04	37.52 ± 6.01	0.209
D_mean_ (Gy)	12.23 ± 9.32	13.31 ± 9.89	0.002

Mean ± SD; P value was calculated by paired t-test. PTV, planning target volume; OARs, organs at risk; FL, functional lung; Dmax, maximum dose; Dmean, mean dose; CI, conformability index; HI, homogeneity index; Vx, volume of receiving ≥ X Gy; 5F-IMRT, five-field fixed-beam functional lung-sparing IMRT planning; 2F-VMAT, two-Arc functional lung-sparing VMAT planning.

## Discussion

In this study, we investigated different strategies and radiotherapy techniques for the preservation of the FL based on 4D-CT ventilation function images in patients with EC. Our results showed that the FL-sparing planning achieved better FL protection compared with the non-sparing planning while satisfying PTV dose coverage and OAR dose limitations. We also demonstrated that the 5F-IMRT had a lower Heart and Lungs irradiated dose but the lowest PTV-CI compared to the 7F-IMRT and 9F-IMRT. The 2F-VMAT had higher PTV-CI and lower Lungs, Heart, and FL dose than the 1F-VMAT. Furthermore, the 2F-VMAT achieved better FL protection compared with the 5F-IMRT.

EC is a commonly diagnosed gastrointestinal tract tumor, and different pathological types have different biological characteristics (1). Radiotherapy plays a unique role in treating EC (especially squamous cell carcinoma), but due to the anatomical location of the esophagus, it inevitably leads to radiation exposure of the lungs. Excessive radiation doses can induce the development of acute radiation pneumonia in the early stages and may progress to pulmonary fibrosis in the late stages, of which the severe cases can even be fatal (28). The risk of RP is further increased when patients combine with smoking, chronic obstructive pulmonary disease, interstitial lung disease, and concurrent chemotherapy, and there is no special treatment drug available (3, 28). Studies have shown that RP was correlated with the irradiated dose and volume of the Lungs (8). The arrival of the 3D-CRT era has improved the tumor target area conformality while reducing the OAR dose than 2D radiotherapy. IMRT is a more advanced technique than 3D-CRT and is currently the main treatment option for EC in clinical practice. However, a meta-analysis showed that although IMRT reduced the mean lung dose compared to 3D-CRT, there was no significant difference in radiation pneumonia in the two groups (5). VMAT is an advanced form of IMRT that provides a higher conformal dose distribution with less treatment time (29). The FL is a subunit with higher functionality identified by functional imaging. It has been demonstrated that the better the functional status of the area, the more sensitive it is to radiation (8). Therefore, reducing the irradiated dose to the FL is necessary by adding dose-limiting conditions and changing the direction of the beams during the radiotherapy planning design (9).

FL imaging modalities involve two aspects, one is lung ventilation function and the other is lung perfusion function. Studies have shown that FL imaging can effectively identify the differences in ventilation and perfusion of lung tissue, and radiation will reduce lung ventilation and perfusion on functional imaging images (10). The 4D-CT is one of the more convenient, high-resolution, and economically low-cost imaging modalities for pulmonary ventilation function. Yet, nuclear medicine imaging has been widely used to assess lung function for a long time, maintaining relative evaluation standards, and SPECT/CT can provide better spatial resolution and 3D anatomical information than history. So, it has been selected as a reference for assessing lung ventilation and perfusion standard (30). Brennan et al. (18) performed a correlation test between 4D-CT ventilation function metrics and PFT parameters in 98 lung cancer patients and showed a good correlation (approximately 0.7). However, PFT reflects overall lung function without distinguishing differences in lung function distribution and has limited sensitivity to early functional changes of disease. Vinogradskiy et al. (19) conducted 4D-CT ventilation and SPECT/CT ventilation function imaging scanning in 15 lung cancer patients simultaneously and showed a correlation coefficient of 0.68 between the two images. A phase 2 clinical trial showed that 4D-CT ventilation function image-guided FL-sparing planning reduced the incidence of RP to 14.9% in lung cancer patients (compared to 25% historical rate), and a phase 3 trial will be performed to validate further (31). Hence, 4D-CT ventilation function imaging integrated into radiotherapy planning is clinically valuable.

Previous studies comparing the dosimetric differences between different radiotherapy techniques in thoracic EC have shown that the 9F-IMRT does not produce lower OAR doses than the 5F-IMRT (32). This is similar to our results that the 5F-IMRT has a lower irradiated dose to the Heart and FL than the 9F-IMRT, and the PTV dose meets the clinical requirements. Gao et al. (33) compared the dosimetric differences between VMAT and IMRT techniques in EC. They found that VMAT reduced the dose of the Lungs and Heart with a similar dose distribution in the tumor target area, which was consistent with our results. FL imaging has been investigated in lung cancer radiotherapy for a long time, and a meta-analysis demonstrated that FL-sparing plans reduced the FL-D_mean_ and FL-V_20_ by 2.2Gy and 4.2%, respectively, when compared with conventional anatomical CT plans, which was also close to our results (reduced 1.4Gy and 4.8%) (10).

The optimally defined threshold for FL has not been determined so far. Most studies were defined as 90% or 70% of the maximum as FL in 4D-CT ventilation function imaging of lung cancer (10). Only Yamamoto et al. (34) utilized the definite thresholds to distinguish three different FL regions. Thus, we evaluated the dosimetric differences of FLs (definite threshold defined) under different protection strategies. Our FL dose limitations were set more strictly because the lungs were irradiated at a lower dose in EC than the lung cancer target area. However, due to the difference in the spatial relationship between FL and target area, some FL dosimetric is challenging to decrease in the FL-sparing planning design. According to our results, the 2F-VMAT is preferentially recommended to obtain better FL-sparing and a shorter treatment time (27). However, it has been reported that with the same IMRT beams, there are differences in the protection of FL with different arrangements, and Wang et al. (23) demonstrated that five-field manually optimized beam IMRT is more protective of the FL than five-field equally spaced beam IMRT. So, manually optimizing beam IMRT may be better when VMAT is unavailable.

There are several limitations in this study. Firstly, a small sample size was included. Secondly, the optimal dose restrictions, weight, and beam arrangement for FL have not achieved widespread consensus, and there may be leeway in FL optimization. Thirdly, 4D-CT only identified the patient’s ventilation function and did not measure lung perfusion function because normal lung function is the process of gas exchange in which air and blood maintain the proper proportion to ensure adequate and effective air exchange. Fourthly, this study only explored the dosimetric differences of different techniques on FL, target areas, and other OARs at the radiotherapy planning level and did not involve actual clinical application in practice, and its value needs to be verified in clinical trials in the future.

## Conclusion

Our study confirms that 4D-CT ventilation function image-based FL protection planning for patients with EC can effectively reduce the FL irradiation dose without compromising target area coverage and other OAR dose limitations. In addition, among different FL protection strategies and radiation treatment techniques, the 7F/9F-IMRT has no better value than the 5F-IMRT except for higher CI, while the 2F-VMAT achieves better PTV conformity and better FL dose reduction.

## Data availability statement

The original contributions presented in the study are included in the article/**Supplementary Material**. Further inquiries can be directed to the corresponding author.

## Ethics statement

The studies involving human participants were reviewed and approved by The Ethics Committee of Affiliated Cancer Hospital and Institute of Guangzhou Medical University. The patients/participants provided their written informed consent to participate in this study. Written informed consent was obtained from the individual(s) for the publication of any potentially identifiable images or data included in this article.

## Author contributions

P-XZ and S-XZ designed the study. HY and G-QZ performed 4D-CT scanning and generated functional lung ventilation images. P-XZ, YZ, and R-HW performed the design of the radiotherapy planning. P-XZ and YZ collected the data. P-XZ and S-XZ wrote and revised the manuscript. All authors contributed to the article and approved the submitted version.

## Acknowledgments

This work was supported by Guangdong Medical Science and Technology Research Fund project (Grant Number: A2021283), Guangzhou Key Medical Discipline Construction Project Found, the Natural Science Foundation of Guangdong Province (No. 2021A1515011329), and the Science and Technology Plan Project of Guangzhou City-school Joint (No. 202201020121). The authors thank all the people who had participated in this study.

## Conflict of interest

The authors declare that the research was conducted in the absence of any commercial or financial relationships that could be construed as a potential conflict of interest.

## Publisher’s note

All claims expressed in this article are solely those of the authors and do not necessarily represent those of their affiliated organizations, or those of the publisher, the editors and the reviewers. Any product that may be evaluated in this article, or claim that may be made by its manufacturer, is not guaranteed or endorsed by the publisher.

## References

[1] Smyth EC Lagergren J Fitzgerald RC Lordick F Shah MA Lagergren P. Oesophageal cancer. Nat Rev Dis Primers (2017) 3:17048. doi: 10.1038/nrdp.2017.48 28748917 PMC6168059

[2] Bray F Ferlay J Soerjomataram I Siegel RL Torre LA Jemal A. Global cancer statistics 2018: GLOBOCAN estimates of incidence and mortality worldwide for 36 cancers in 185 countries. CA Cancer J Clin (2018) 68:394–424. doi: 10.3322/caac.21492 30207593

[3] Du F Qiang W Wei W Yingjie Z Zhenxiang L Jianbin L. Analysis of related factors of radiation pneumonitis after radiotherapy for thoracic segment esophageal cancer. Chin Radiol Med Prot (2020) 40:832–9. doi: 10.3760/cma.j.issn.0254-5098.2020.11.004

[4] Branch of Radiation Oncology Physicians of Chinese Medical Doctor Association Chinese medical association branch of radiotherapy therapy Cancer Radiotherapy Committee of Chinese Anti-Cancer Association. Chinese Esophageal cancer radiotherapy guidelines (2020 version). J Int Oncol (2020) 47:641–55. doi: 10.3760/cma.j.cn371439-20201015-00095

[5] Xu D Li G Li H Jia F. Comparison of IMRT versus 3D-CRT in the treatment of esophagus cancer. Med (Baltimore) (2017) 96:e7685. doi: 10.1097/MD.0000000000007685 PMC5626151 28767597

[6] Wijsman R Dankers F Troost EGC Hoffmann AL van der Heijden EHFM de Geus-Oei L. Comparison of toxicity and outcome in advanced stage non-small cell lung cancer patients treated with intensity-modulated (chemo-)radiotherapy using IMRT or VMAT. Radiother Oncol (2017) 122:295–9. doi: 10.1016/j.radonc.2016.11.015 27914680

[7] De Ruysscher D Niedermann G Burnet NG Siva S Lee AWM Hegi-Johnson F. Radiotherapy toxicity. Nat Rev Dis Primers (2019) 5:13. doi: 10.1038/s41572-019-0064-5 30792503

[8] Siva S Hardcastle N Kron T Bressel M Callahan J MacManus MP. Ventilation/Perfusion positron emission tomography–based assessment of radiation injury to lung. Int J Radiat Oncol Biol Phys (2015) 93:408–17. doi: 10.1016/j.ijrobp.2015.06.005 26275510

[9] Partridge M Yamamoto T Grau C Høyer M Muren LP. Imaging of normal lung, liver and parotid gland function for radiotherapy. Acta Oncol (2010) 49:997–1011. doi: 10.3109/0284186X.2010.504735 20831488

[10] Bucknell NW Hardcastle N Bressel M Hofman MS Kron T Ball D. Functional lung imaging in radiation therapy for lung cancer: A systematic review and meta-analysis. Radiother Oncol (2018) 129:196–208. doi: 10.1016/j.radonc.2018.07.014 30082143

[11] Faught AM Miyasaka Y Kadoya N Castillo R Castillo E Vinogradskiy Y. Evaluating the toxicity reduction with computed tomographic ventilation functional avoidance radiation therapy. Int J Radiat Oncol Biol Phys (2017) 99:325–33. doi: 10.1016/j.ijrobp.2017.04.024 PMC5605907 28871982

[12] Farr KP Kallehauge JF Møller DS Khalil AA Kramer S Bluhme H. Inclusion of functional information from perfusion SPECT improves predictive value of dose-volume parameters in lung toxicity outcome after radiotherapy for non-small cell lung cancer: A prospective study. Radiother Oncol (2015) 117:9–16. doi: 10.1016/j.radonc.2015.08.005 26303012

[13] Vinogradskiy Y Rusthoven CG Schubert L Jones B Faught A Castillo R. Interim analysis of a two-institution, prospective clinical trial of 4DCT-ventilation-based functional avoidance radiation therapy. Int J Radiat Oncol Biol Phys (2018) 102:1357–65. doi: 10.1016/j.ijrobp.2018.07.186 PMC6919556 30353873

[14] Functional lung avoidance SPECT-guided radiation therapy of lung cancer - full text view - ClinicalTrials.gov (2022). Available at: https://clinicaltrials.gov/ct2/show/NCT04676828.

[15] Wilson JM Partridge M Hawkins M. The application of functional imaging techniques to personalise chemoradiotherapy in upper gastrointestinal malignancies. Clin Oncol (R Coll Radiol) (2014) 26:581–96. doi: 10.1016/j.clon.2014.06.009 PMC4150923 24998430

[16] Yamamoto T Kabus S Lorenz C Mittra E Hong JC Chung M. Pulmonary ventilation imaging based on 4-dimensional computed tomography: Comparison with pulmonary function tests and SPECT ventilation images. Int J Radiat Oncol Biol Phys (2014) 90:414–22. doi: 10.1016/j.ijrobp.2014.06.006 PMC8112248 25104070

[17] Waxweiler T Schubert L Diot Q Faught A Stuhr K Castillo R. A complete 4DCT-ventilation functional avoidance virtual trial: Developing strategies for prospective clinical trials. J Appl Clin Med Phys (2017) 18:144–52. doi: 10.1002/acm2.12086 PMC5689844 28436107

[18] Brennan D Schubert L Diot Q Castillo R Castillo E Guerrero T. Clinical validation of 4-dimensional computed tomography ventilation with pulmonary function test data. Int J Radiat Oncol Biol Phys (2015) 92:423–9. doi: 10.1016/j.ijrobp.2015.01.019 PMC4431937 25817531

[19] Vinogradskiy Y Koo PJ Castillo R Castillo E Guerrero T Gaspar LE. Comparison of 4-dimensional computed tomography ventilation with nuclear medicine ventilation-perfusion imaging: A clinical validation study. Int J Radiat Oncol Biol Physs (2014) 89:199–205. doi: 10.1016/j.ijrobp.2014.01.009 PMC4121964 24725702

[20] Pinder-Arabpour A Jones B Castillo R Castillo E Guerrero T Goodman K. Characterizing spatial lung function for esophageal cancer patients undergoing radiation therapy. Int J Radiat Oncol Biol Phys (2019) 103:738–46. doi: 10.1016/j.ijrobp.2018.10.024 PMC7063843 30612962

[21] Latifi K Forster KM Hoffe SE Dilling TJ van Elmpt W Dekker A. Dependence of ventilation image derived from 4D CT on deformable image registration and ventilation algorithms. J Appl Clin Med Phys (2013) 14:4247. doi: 10.1120/jacmp.v14i4.4247 23835389 PMC5714535

[22] Klein S Staring M Murphy K Viergever MA Pluim JPW. Elastix: A toolbox for intensity-based medical image registration. IEEE T Med Imaging (2010) 29:196–205. doi: 10.1109/TMI.2009.2035616 19923044

[23] Wang R Zhang S Yu H Lin S Zhang G Tang R. Optimal beam arrangement for pulmonary ventilation image-guided intensity-modulated radiotherapy for lung cancer. Radiat Oncol (2014) 9:184. doi: 10.1186/1748-717X-9-184 25127899 PMC4141960

[24] Reinhardt JM Ding K Cao K Christensen GE Hoffman EA Bodas SV. Registration-based estimates of local lung tissue expansion compared to xenon CT measures of specific ventilation. Med Image Anal (2008) 12:752–63. doi: 10.1016/j.media.2008.03.007 PMC2692217 18501665

[25] Yijun AN Biao Z Yutao Z Liqiu HE Kewei T Yi Y. VMAT versus TOMO in dosimetric parameters for treatment of middle thoracic esophageal cancer. Acad J Chin PLA Med Sch (2018) 39:312–5. doi: 10.3969/j.issn.2095-5227.2018.04.011

[26] Zhou P. Research on protection of lung functions in patients with esophageal cancer radiotherapy based on 4D-CT ventilation images. Guangzhou Medical University (2022).

[27] Yu D Bai Y Feng Y Wang L Yun W Li X. Which bone marrow sparing strategy and radiotherapy technology is most beneficial in bone marrow-sparing intensity modulated radiation therapy for patients with cervical cancer? Front Oncol (2020) 10:554241. doi: 10.3389/fonc.2020.554241 33392067 PMC7773663

[28] Hanania AN Mainwaring W Ghebre YT Hanania NA Ludwig M. Radiation-induced lung injury: Assessment and management. Chest (2019) 156:150–62. doi: 10.1016/j.chest.2019.03.033 PMC8097634 30998908

[29] Shao Y Chen H Wang H Duan Y Feng A Huang Y. Investigation of predictors to achieve acceptable lung dose in T-shaped upper and middle esophageal cancer with IMRT and VMAT. Front Oncol (2021) 11:735062. doi: 10.3389/fonc.2021.735062 34692508 PMC8529030

[30] Bahig H Campeau M Lapointe A Bedwani S Roberge D de Guise J. Phase 1-2 study of dual-energy computed tomography for assessment of pulmonary function in radiation therapy planning. Int J Radiat Oncol Biol Phys (2017) 99:334–43. doi: 10.1016/j.ijrobp.2017.05.051 28871983

[31] Vinogradskiy Y Castillo R Castillo E Schubert L Jones BL Faught A. Results of a multi-institutional phase 2 clinical trial for 4DCT-ventilation functional avoidance thoracic radiation therapy. Int J Radiat Oncol Biol Phys (2021) 112(4):986–95. doi: 10.1016/j.ijrobp.2021.10.147 PMC8863640 34767934

[32] Yan-li Y Bao-sheng LI Yong Y Jin-hu C Tao S Hong-fu S. Dosimetric comparison of three-dimensional conformal radiotherapy, intensity-modulated radiotherapy and RapidArc in treatment of thoracic esophageal cancer. Chin Radiol Med Prot (2012) 32:65–9. doi: 10.3760/cma.j.issn.0254-5098.2012.01.016

[33] Gao M Li Q Ning Z Gu W Huang J Mu J. Dosimetric comparison between step-shoot intensity-modulated radiotherapy and volumetric-modulated arc therapy for upper thoracic and cervical esophageal carcinoma. Med Dosim (2016) 41:131–5. doi: 10.1016/j.meddos.2015.10.007 26920244

[34] Yamamoto T Kabus S von Berg J Lorenz C Keall PJ. Impact of four-dimensional computed tomography pulmonary ventilation imaging-based functional avoidance for lung cancer radiotherapy. Int J Radiat Oncol Biol Phys (2011) 79:279–88. doi: 10.1016/j.ijrobp.2010.02.008 20646852

