# Supplementary material for: Different functional lung-sparing strategies and radiotherapy techniques for patients with esophageal cancer
Source: Front Oncol. 2022 Aug 26;12:898141. doi: 10.3389/fonc.2022.898141 (PMC9459335; doi:10.3389/fonc.2022.898141)
Supplement: Supplementary file 1 [file DataSheet_1.docx]

**Table S1**. Dosimetric parameters comparison for PTV and OARs in different FL-sparing IMRT planning.

| OARs | 5F-IMRT | 7F-IMRT | 9F-IMRT | P value | | |
| --- | --- | --- | --- | --- | --- | --- |
|  |  |  |  | 5F vs. 7F | 5F vs. 9F | 7F vs. 9F |
| PTV |  |  |  |  |  |  |
| D_max_ (Gy) | 58.19 ± 8.83 | 58.46 ± 8.67 | 57.90 ± 8.72 | 0.319 | 0.414 | 0.160 |
| D_mean_ (Gy) | 52.54 ±7.99 | 52.91 ± 8.72 | 52.61 ± 8.17 | 0.207 | 0.649 | 0.262 |
| CI | 0.647 ± 0.106 | 0.670 ± 0.103 | 0.681 ± 0.087 | 0.008 | 0.042 | 0.360 |
| HI | 1.152 ± 0.076 | 1.148 ± 0.083 | 1.138 ± 0.067 | 0.374 | 0.074 | 0.323 |
| FL |  |  |  |  |  |  |
| D_mean_ (Gy) | 6.04 ± 2.32 | 6.05 ± 2.27 | 5.89 ± 2.15 | 0.940 | 0.163 | 0.090 |
| V_5_ (%) | 37.61 ± 14.95 | 37.92 ± 15.27 | 37.26 ± 15.28 | 0.490 | 0.659 | 0.431 |
| V_10_ (%) | 16.55 ± 7.65 | 16.41 ± 7.35 | 15.31 ± 6.69 | 0.830 | 0.117 | 0.076 |
| V_20_ (%) | 6.52 ± 3.76 | 6.43 ± 3.71 | 6.21 ± 3.95 | 0.662 | 0.265 | 0.362 |
| V_30_ (%) | 3.61 ± 2.53 | 3.52 ± 2.51 | 3.24 ± 2.48 | 0.675 | 0.043 | 0.067 |
| Lungs |  |  |  |  |  |  |
| D_mean_ (Gy) | 9.18 ± 2.63 | 9.36 ± 2.66 | 9.11 ± 2.60 | 0.017 | 0.485 | 0.012 |
| V_5_ (%) | 50.44 ± 13.05 | 50.77 ± 12.69 | 50.36 ± 12.08 | 0.461 | 0.904 | 0.342 |
| V_10_ (%) | 30.51 ± 8.82 | 31.85 ± 8.58 | 30.45 ± 8.30 | 0.053 | 0.921 | 0.011 |
| V_20_ (%) | 13.71 ± 5.43 | 14.09 ± 6.03 | 13.70 ± 6.06 | 0.156 | 0.993 | 0.474 |
| V_30_ (%) | 6.86 ± 3.56 | 6.71 ± 3.57 | 6.51 ± 3.47 | 0.470 | 0.233 | 0.253 |
| Heart |  |  |  |  |  |  |
| D_mean_ (Gy) | 17.38 ± 10.30 | 17.63 ± 10.55 | 17.45 ± 10.45 | 0.210 | 0.728 | 0.203 |
| V_5_ (%) | 60.88 ± 35.20 | 62.04 ± 35.11 | 61.88 ± 35.14 | 0.004 | 0.035 | 0.459 |
| V_10_ (%) | 53.10 ± 34.06 | 55.33 ± 34.48 | 54.65 ± 34.02 | 0.007 | 0.070 | 0.209 |
| V_20_ (%) | 38.34 ± 25.07 | 40.06 ± 27.59 | 39.04 ± 27.32 | 0.108 | 0.541 | 0.221 |
| V_30_ (%) | 23.80 ± 16.08 | 23.39 ± 16.55 | 23.06 ± 16.58 | 0.456 | 0.248 | 0.279 |
| V_40_ (%) | 13.61 ± 11.30 | 13.14 ± 11.04 | 13.07 ± 11.31 | 0.312 | 0.346 | 0.844 |
| Spinal Cord |  |  |  |  |  |  |
| D_max_ (Gy) | 40.01 ± 4.04 | 40.04 ± 3.63 | 39.89 ± 3.88 | 0.956 | 0.799 | 0.642 |
| D_mean_ (Gy) | 12.23 ± 9.32 | 12.66 ± 9.15 | 12.63 ± 8.60 | 0.121 | 0.243 | 0.898 |

Key: mean ± SD; statistical analysis was calculated by paired t test. PTV: planning target volume; OARs: organs at risk; FL: functional lung; D_max_: maximum dose; D_mean_: mean dose; CI: conformability index; HI: homogeneity index; Vx: volume of receiving ≥ X Gy; 5F-IMRT: five-field fixed-beam functional lung-sparing IMRT planning; 7F-IMRT: seven-field fixed-beam functional lung-sparing IMRT planning; 9F-IMRT: nine-field fixed-beam functional lung-sparing IMRT planning;

**Table S2**. Dosimetric parameters comparison for PTV and OARs in different FL-sparing VMAT planning.

| OARs | 1F-VMAT | 2F-VMAT | P value |
| --- | --- | --- | --- |
| PTV |  |  |  |
| D_max_ (Gy) | 57.02 ± 8.40 | 56.77 ± 8.75 | 0.238 |
| D_mean_ (Gy) | 52.48 ± 8.01 | 52.43 ± 7.96 | 0.394 |
| CI | 0.711 ± 0.100 | 0.711 ± 0.113 | 0.997 |
| HI | 1.137 ± 0.073 | 1.131 ± 0.071 | 0.048 |
| FL |  |  |  |
| D_mean_ (Gy) | 5.73 ± 2.08 | 5.64 ± 2.01 | 0.046 |
| V_5_ (%) | 35.09 ± 13.87 | 34.78 ± 13.32 | 0.489 |
| V_10_ (%) | 14.07 ± 6.23 | 13.27 ± 5.99 | 0.041 |
| V_20_ (%) | 6.28 ± 3.74 | 5.99 ± 3.57 | 0.113 |
| V_30_ (%) | 3.22 ± 2.23 | 3.22 ± 2.29 | 0.960 |
| Lungs |  |  |  |
| D_mean_ (Gy) | 8.79 ± 2.51 | 8.69 ± 2.43 | 0.069 |
| V_5_ (%) | 46.69 ± 10.73 | 46.03 ± 9.80 | 0.164 |
| V_10_ (%) | 27.91 ± 7.87 | 27.07 ± 7.52 | 0.013 |
| V_20_ (%) | 13.56 ± 5.77 | 13.20 ± 5.62 | 0.100 |
| V_30_ (%) | 6.35 ± 3.24 | 6.46 ± 3.27 | 0.595 |
| Heart |  |  |  |
| D_mean_ (Gy) | 17.30 ± 10.58 | 17.15 ± 10.34 | 0.281 |
| V_5_ (%) | 61.38 ± 35.45 | 61.51 ± 35.53 | 0.528 |
| V_10_ (%) | 54.38 ± 34.15 | 53.89 ± 33.48 | 0.203 |
| V_20_ (%) | 40.67 ± 28.11 | 39.56 ± 26.96 | 0.022 |
| V_30_ (%) | 23.30 ± 16.69 | 22.59 ± 15.86 | 0.205 |
| V_40_ (%) | 11.77 ± 9.91 | 12.17 ± 10.41 | 0.160 |
| Spinal cord |  |  |  |
| D_max_ (Gy) | 39.80 ± 4.48 | 37.52 ± 6.01 | 0.274 |
| D_mean_ (Gy) | 13.28 ± 9.64 | 13.31 ± 9.89 | 0.790 |

Key: mean ± SD; P value was calculated by paired t test. PTV: planning target volume; OARs: organs at risk; FL: functional lung; D_max_: maximum dose; D_mean_: mean dose; CI: conformability index; HI: homogeneity index; Vx: volume of receiving ≥ X Gy; 1F-VMAT: one-Arc functional lung-sparing VMAT planning. 2F-VMAT: two-Arc functional lung-sparing VMAT planning.
